# Perceived ease of use, perceived enjoyment, and continuance intention as predictors of medical students’ well-being in hybrid learning: an extended TAM approach

**DOI:** 10.3389/fpsyg.2025.1704678

**Published:** 2026-01-05

**Authors:** Qiumei Wang, Hui Zhan, Yu Liang, Peilong Li, Lin Ai

**Affiliations:** 1 Youjiang Medical University for Nationalities, Baise, China; 2 Patton College of Education, Ohio University, Athens, OH, United States

**Keywords:** hybrid learning, students’ well-being, perceived ease of use, perceived enjoyment, continuance intention, medical education

## Abstract

**Introduction:**

Although hybrid learning has transformed educational environments by enhancing student engagement and learning outcomes, challenges related to medical students’ well-being have emerged, particularly with the application of the technology acceptance model (TAM).

**Methods:**

To explore how technology-related factors, such as perceived ease of use, perceived enjoyment, and continuance intention, impact medical students’ well-being, a set of questionnaires was distributed to 337 randomly selected undergraduate students from three medical universities in Guangxi, China.

**Results:**

The results of the partial least squares structural equation modeling (PLS-SEM) indicated that the ease of use and perceived enjoyment of hybrid learning systems had a significant, direct, and positive impact on students’ well-being. This impact was also mediated by students’ intentions to continue utilizing the system in the learning process.

**Discussion:**

Both perceived ease of use and perceived enjoyment significantly influenced students’ continuance intention, which positively affected their well-being. Perceived ease of use significantly affects students’ well-being, much more than enjoyment, emphasizing the importance of user-friendly interfaces over purely enjoyable features in hybrid learning platforms for medical students. By positioning well-being as a central outcome, this study extends the explanatory power of the TAM beyond acceptance and satisfaction, offering theoretical insights and practical guidance for designing hybrid learning systems that support both academic achievement and psychological health.

## 1 Introduction

With the advancement of Internet, hybrid learning has transformed significantly since the early 2000s, moving from traditional classroom instruction to a combination of online and in-person learning methods. This methodology has improved student learning and critical thinking by enabling colleges worldwide to provide engaging and interactive learning content (Wei and Hu, 2018). Through collaborative activities and project-based learning, hybrid learning fosters active student participation and creates a dynamic learning environment (Li, 2022). Research conducted in several nations has shown that hybrid learning can lower dropout rates and boost motivation, especially during the COVID-19 pandemic, prompting a swift transition to online formats (Horita et al., 2021; Marzoli et al., 2021; Shin and Hickey, 2021). Driven by the need for flexibility and efficient use of technology, along with the COVID-19 pandemic, hybrid learning has become increasingly popular in medical education worldwide (Raes, 2022).

However, the shift from traditional learning to hybrid learning has also brought to light some significant challenges, such as a decline in student engagement, lower academic performance (Maqableh and Alia, 2021), and a decrease in students’ well-being (Kemp, 2023). In particular, dental students experienced varying levels of depression in hybrid learning environments during the pandemic (Orozco et al., 2023). These issues highlight the crucial need to incorporate medical students’ well-being into hybrid learning frameworks, as it significantly affects their academic performance and motivation (Sánchez-Cruzado et al., 2021).

From a theoretical perspective, the traditional technology acceptance model (TAM), which primarily focuses on perceived ease of use and usefulness to predict technology acceptance and usage (Davis, 1989), appears to be insufficient to fully capture the complexities of technology acceptance, especially in medical education settings. The TAM has been expanded to include various external variables, such as perceived convenience (Hsu and Chang, 2013), technical support (Sánchez and Hueros, 2010), and perceived playfulness (Padilla-Meléndez et al., 2013). It has been adapted to various professional contexts, including integration with the demands-resources theory, to explore employees’ well-being in the workplace (Shamsi et al., 2021). Furthermore, an emotional TAM has been extended to examine the factors influencing well-being in online environments (Lu et al., 2019). These emotional- or well-being-extended TAM studies have mainly been conducted in workplace settings, general Internet use contexts, or fully online learning environments, often viewing well-being as a secondary or indirect outcome rather than the primary dependent variable. Moreover, these studies usually focus on either emotional or post-adoption constructs and rarely consider both affective and continuance-related constructs within a single framework. However, few studies have focused on extending the TAM to address students’ well-being in hybrid learning environments, particularly in the context of medical education.

**Figure 1:**
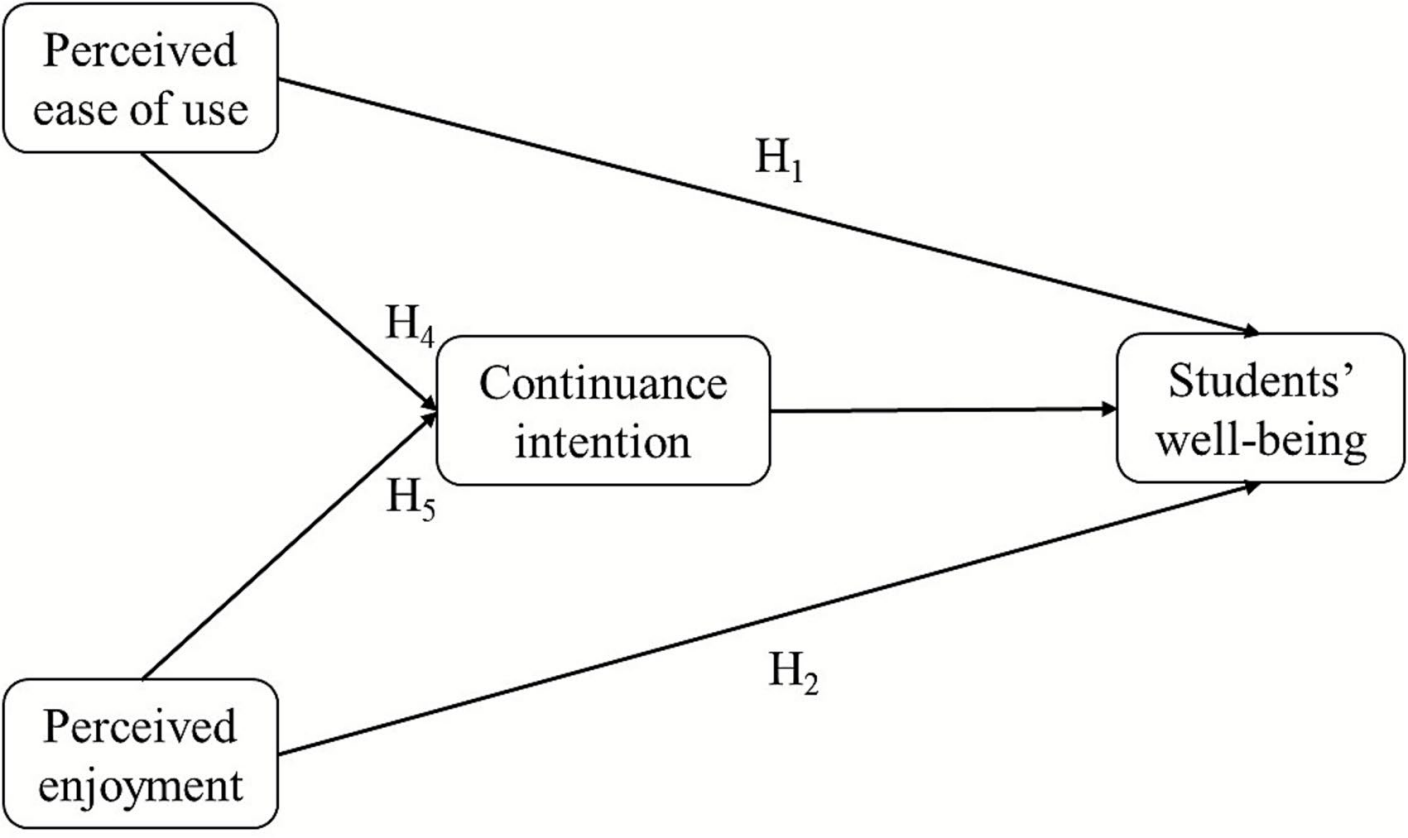
Extended TAM for medical students’ well-being in hybrid learning environments.

Given the critical importance of incorporating medical students’ well-being into a hybrid learning environment and the need to develop a modified well-being TAM framework in hybrid learning, this study aimed to develop a model in which perceived ease of use and perceived enjoyment are hypothesized to influence students’ continuance intention, with medical students’ well-being as the primary outcome in hybrid learning environments. As shown in Figure 1, perceived ease of use and perceived enjoyment were modeled as exogenous variables, continuance intention served as a mediating variable, and well-being was identified as the final endogenous outcome. This framework addresses three key limitations found in previous research: (1) Well-being is rarely considered a direct outcome of hybrid learning technologies; (2) continuance intention has seldom been examined as the pathway linking ease of use and enjoyment to well-being; and (3) medical education contexts with their unique stressors have received limited attention. This modified framework aims to guide the development of hybrid learning environments that not only enhance learning outcomes but also support medical students’ mental health. These findings provide valuable insights for technology developers, medical educators, and policymakers in creating inclusive and supportive hybrid learning strategies that promote long-term well-being for students in medical education settings.

## 2 Literature review

### 2.1 Well-being

The concept of well-being has been widely explored in various contexts, particularly in relation to how individuals evaluate their overall life satisfaction and happiness (Deci and Ryan, 2008). In the context of technology and digital interactions, well-being has gained new dimensions that are pivotal to understanding user interaction and the broader impact of technological environments.

Well-being in digital environments is conceptualized as the aspects of an individual’s physical, psychological, and social health that they encounter when interacting with technology (Orben and Przybylski, 2019). These aspects include engagement, positive affect, reduced tension, satisfaction, and an improved overall quality of life (Webster and Martocchio, 1992). A comprehensive understanding of these aspects is crucial for technology design and adoption, emphasizing the necessity of considering how technology influences users’ holistic experiences and outcomes (Phaekwamdee et al., 2022).

Students’ well-being is particularly important in educational settings. In positive psychology, students’ well-being is often described using the PERMA model, which includes positive emotions, engagement, relationships, meaning, and accomplishments (Kern et al., 2014; Seligman, 2011). This framework emphasizes that well-being is not only the absence of distress but also the presence of positive emotions, sustained engagement in activities, supportive relationships, a sense of purpose, and perceived accomplishment. Studies have shown that students with higher levels of well-being tend to display greater persistence, effective problem-solving abilities, and intrinsic motivation (Kern et al., 2014). Furthermore, it has been highlighted that students’ sustained well-being is strongly correlated with their academic success (Kaya and Erdem, 2021). Therefore, for medical students who often face intensive workloads and stress, understanding how technology-supported hybrid learning environments affect their well-being is critical.

### 2.2 Perceived ease of use and well-being

As a key concept of the TAM, perceived ease of use is defined as the extent to which a user believes that using a specific technology will be effortless (Davis, 1989). Within the TAM, perceived ease of use, together with perceived usefulness, shapes a user’s attitude toward adopting the technology, ultimately influencing both their intention to use it and their actual usage of the system. This model also posits that an easy-to-use interface not only enhances user satisfaction but also promotes continuous user engagement with technology.

Research across various fields has highlighted the significance of perceived ease of use in shaping user behavior and well-being. For instance, perceived ease of use has been found to significantly impact technology acceptance and work engagement among employees, suggesting a link between ease of use and overall well-being in the workplace (Shamsi et al., 2021). Moreover, research in the field of smart-connected objects has shown that user well-being gradually improves through various stages of technology adoption, influenced by perceived ease of use, perceived usefulness, and social image (Attié and Meyer-Waarden, 2020).

Similarly, in the context of e-learning, it has been discovered that the ease of use of e-learning platforms has a direct impact on student satisfaction (Alqahtani et al., 2022) and well-being (Ficapal-Cusí et al., 2024). Researchers have further elaborated on this concept in online education, illustrating how perceived ease of use, along with interactive and engaging content, significantly improves students’ well-being in classrooms (Xiao et al., 2023). They noted that the quality and pleasure of audio content can enhance the positive effects of teacher–student interactions on overall well-being. Although several studies have explored the relationship between perceived ease of use and students’ well-being, only a few have been focused on a hybrid learning environment in medical education.

*H1. Perceived ease of use predicts students’ well-being in hybrid learning.*

### 2.3 Perceived enjoyment and well-being

Perceived enjoyment refers to the pleasure one feels when using a system, regardless of how it affects their performance (Davis, 1989). This concept is recognized as a key contributor to user engagement and satisfaction in both technological and educational contexts (Brown and Venkatesh, 2005). Within the PERMA framework, enjoyment is a positive emotion that links technology-related experiences to broader indicators of student well-being. Therefore, in this study, perceived enjoyment was treated as an independent predictor of learners’ affective responses to hybrid learning systems.

Perceived enjoyment not only enhances job satisfaction in the workplace but also significantly contributes to overall well-being when individuals feel connected to their coworkers and competent in their roles (Frederick and Lazzara, 2020). This positive impact of perceived enjoyment also extends to the educational field, particularly in e-learning environments, where perceived enjoyment was found to positively mediate the relationship between self-management in e-learning and students’ well-being (Ficapal-Cusí et al., 2024).

Despite these insights, little is known about the impact of perceived enjoyment on well-being in the context of hybrid learning. Recent studies have explicitly called for more research into how perceived enjoyment relates to students’ well-being across different learning environments (Ficapal-Cusí et al., 2024).

*H2. Perceived enjoyment predicts students’ well-being in hybrid learning.*

### 2.4 Continuance of intention and well-being

Continuance intention, defined as the decision to persistently use or reuse a system, is a crucial concept in technology acceptance and user behavior (Davis, 1989). It is closely linked to user satisfaction, enjoyment, attitude, and perceived usefulness (Davis, 1989). Recent studies have found that continuing to use information technology after its initial adoption is essential for its long-term viability and success, particularly in learning environments (Taghizadeh et al., 2022; Zhang and Xu, 2020; Zhu et al., 2022). From an information systems continuance perspective, continuance intention can be regarded as a post-adoption construct that mediates the effects of users’ beliefs about a system (e.g., perceived ease of use and perceived enjoyment) on long-term outcomes, such as well-being, by shaping the extent to which they continue to use the system (e.g., Bhattacherjee, 2001).

An empirical study involving 615 Internet users in the US revealed a positive relationship between the continuance intention to use the Internet, well-being, and perceived value (Lu et al., 2019). Additionally, research conducted in the context of massive multiplayer online games has shown that players’ intentions to play are generally associated with positive feelings and improved well-being, even when faced with challenges or defeats (Linares et al., 2021). This suggests that the motivation to improve and continue participating can lead to psychological benefits, supporting the notion that continuance intention can significantly impact users’ well-being.

*H3. Continuance intention predicts students’ well-being in hybrid learning.*

### 2.5 Perceived ease of use, perceived enjoyment, and continuance intention

The TAM posits that perceived ease of use and perceived usefulness fundamentally shape an individual’s attitude toward technology usage, thereby influencing their behavioral intentions and actual usage patterns (Davis, 1989). Subsequent extensions of this model, such as the Unified Theory of Acceptance and Use of Technology, have further highlighted the role of affective variables, including enjoyment, in predicting technology-related intentions (Venkatesh et al., 2003). In the present study, perceived ease of use and perceived enjoyment were conceptualized as key antecedents of continuance intention in hybrid learning.

Empirical findings on the relationship between perceived ease of use and continuance intention are mixed but generally supportive. In the context of non-fungible token trading platforms, perceived ease of use significantly influences the intention to continue using the platform (Sun, 2024). Similarly, research on Massive Open Online Courses has identified perceived ease of use as a key factor affecting learners’ continuance intention, mediated by its interaction with teaching presence and task-technology fit (Kim and Song, 2022). Additionally, a study on smartwatches demonstrated that ease of use significantly enhances user satisfaction, which, in turn, affects continuance intention (Erkent et al., 2021). Conversely, some studies have reported that perceived ease of use does not significantly influence continuance intention (Han and Sa, 2022; Linares et al., 2021).

Despite these inconsistencies, research on hybrid learning environments continues to emphasize the importance of ease of use. A recent study found that students are more likely to choose blended learning systems that are easy to use (Laifa et al., 2023).

*H4. Perceived ease of use predicts continuance intention in hybrid learning.*

In the context of physical exercise, researchers have found that enjoyment significantly enhances exercise persistence (Teixeira et al., 2022). Furthermore, another study discovered that enjoyment not only positively impacts persistence in exercise routines but also predicts academic persistence (Brubacher and Silinda, 2019; Rodrigues et al., 2020).

The influence of perceived enjoyment on continuance intention has been specifically validated in the realms of mobile and hybrid learning (Li et al., 2025). Research has confirmed that perceived enjoyment significantly and positively affects users’ continuance intentions in hybrid learning applications (Linares et al., 2021). This finding aligns with research on mobile learning, which demonstrated that students’ satisfaction and greater intentions to use a mobile learning platform were directly influenced by the enjoyment they experienced during the learning process (Chao, 2019).

*H5. Perceived enjoyment predicts continuance intention in hybrid learning.*

## 3 Methodology

This study employed a quantitative cross-sectional survey design.

### 3.1 Sampling and data collection

The target population consisted of undergraduate students from three medical universities in Guangxi, China. The sample size was calculated based on the guidelines for partial least squares structural equation modeling (PLS-SEM) multivariate analysis. Following the sample size recommendations (Hair et al., 2022), the required sample size for this model, considering five paths (α = 5%, R^2^ = 0.10, and a power of 80%), was calculated as n = 120. To obtain the target sample size, undergraduate students were selected using a proportionate technique (stratified sampling) in order to ensure equal representation of the population. The total student populations at the three medical universities were 7,271, 7,450, and 9,804, with minimum sample targets of 36, 36, and 48 students, respectively. In the second stage, a single-stage cluster sampling technique was employed. Since classrooms are natural clusters in educational surveys (Lohr, 2021), and given that the number of students in a single elective class across the three universities ranged from 22 to 115, this study randomly selected two elective course classes that were open to all undergraduate students, regardless of their discipline or academic year. To enhance the robustness of the findings and account for potential non-responses, two elective course classes were randomly selected with the assistance of teaching administrators from each university.

Participants voluntarily participated in the survey by scanning a QR code that provided access to the questionnaire. Before administering the survey, lecturers of the selected elective courses provided a brief explanation of the research objectives and emphasized that participation was entirely voluntary and confidential. During the data collection, a total of 337 completed questionnaires were collected. There were no missing values in the dataset. The demographic breakdown of the sample included 240 female students and 97 male students, with the majority of respondents aged 19–20 years (n = 311, 92.3%).

### 3.2 Measures

The questionnaire comprised two sections. The first section collected demographic information, including participants’ sex, major, and age. The second section included items that measured the four study variables. All items ranged from 1 (strongly disagree) to 5 (strongly agree). Before distribution, the English version of the instrument was validated by three experts in education research and translated by two experts in English–Chinese translation. The measurement scales are presented in Appendix A.

Well-being was measured using three items adapted from the study satisfaction component of the Satisfaction with Life and Studies Scale (Holm-Hadulla and Hofmann, 2007). Several studies have used satisfaction to assess study-related well-being (e.g., Berger et al., 2015; Reschke et al., 2024). The original questions (“How satisfied are you with your current academic achievements?,” “How satisfied are you with your current study situation?,” and “How satisfied are you with your general study conditions?”) were rephrased as first-person statements and contextualized for this study (e.g., “I am satisfied with my current academic achievements in hybrid learning,” “I am satisfied with my current study situation in hybrid learning,” and “I am satisfied with my general study conditions in hybrid learning”).

Perceived ease of use was measured using four items adapted from Venkatesh (2000). Perceived ease of use refers to “an assessment of the mental effort involved in using a system” (Venkatesh, 2000, p. 697). To ensure that the measurement scales were contextually appropriate for this study, the phrase “hybrid learning” was added to the four items. For instance, the original item, “My interaction with the system is clear and understandable,” was revised to “My interaction with the hybrid learning system is clear and understandable.” While Venkatesh (2000) employed a seven-point Likert scale, the response format in this study was revised to a five-point Likert scale to ensure consistency with the measurements of other variables.

Perceived enjoyment was measured using four items adapted from Linares et al. (2021). In their study, perceived enjoyment captured the extent to which the system was enjoyable, fun, exciting, and pleasant. To ensure that the measurement scale was contextually appropriate for this study, all items were adapted to the hybrid learning context by replacing “the online game” with “hybrid learning.” For example, the original item “Using the online game is enjoyable” was revised to “Using hybrid learning is enjoyable.”

Continuance intention was measured using two items adapted from Linares et al. (2021). Continuance intention reflects the learners’ intention to continue engaging with the system over time. To ensure that the measurement scale was contextually appropriate for this study, the phrase “hybrid learning” was substituted for “the online game” in the original items. For instance, the original item “I intend to continue using the online game in the future” was revised to “I intend to continue using hybrid learning in the future.”

## 4 Results

The collected data were analyzed using SmartPLS 4.0 (SmartPLS GmbH, Germany), a software tool designed to address complex modeling challenges. This software provides users with an intuitive graphical interface and powerful algorithms for reflective and formative measurement analyses. Using this method, researchers can develop theoretical models by analyzing the relationships between observed and latent variables using path modeling and bootstrapping techniques.

### 4.1 Measurement model assessment

In PLS-SEM, the measurement model was evaluated to confirm the reliability and validity of the constructs. Indicator reliability was achieved by an indicator’s outer loadings higher than 0.70 (Hair et al., 2022). Table 1 shows that the outer loading values of the remaining indicators are greater than 0.792, achieving an indicator reliability threshold of 0.7. Internal consistency reliability was assessed using Cronbach’s alpha and composite reliability (CR) values between 0.70 and 0.95 (Hair et al., 2022). As shown in Table 1, the alpha and CR values of this model were greater than 0.854, indicating excellent internal consistency.

**Table 1:** Reliability and convergent validity of the measurement model.

Indicator	Outer loading	Alpha	CR	AVE
Perceived ease of use		0.854	0.856	0.696
PEU1	0.792			
PEU2	0.855			
PEU3	0.860			
PEU4	0.829			
Perceived enjoyment		0.957	0.958	0.887
POE1	0.913			
POE2	0.958			
POE3	0.950			
POE4	0.946			
Continuance intention		0.943	0.947	0.946
CON1	0.971			
CON2	0.975			
Students’ well-being		0.894	0.895	0.826
UWB1	0.914			
UWB2	0.912			
UWB3	0.900			

Convergent validity was evaluated using the extracted outer loadings and the average variance extracted (AVE). According to Hair et al. (2022), an AVE value higher than 0.50 and an outer loading higher than 0.70 indicate good convergent validity of a model. As shown in Table 1, the AVE values for all constructs were higher than 0.696, thereby confirming the establishment of convergent validity.

Subsequently, the researcher assessed the Heterotrait–Monotrait (HTMT) ratio to confirm discriminant validity (Hair et al., 2022). Discriminant validity was established when the HTMT value was below 0.90 (Hair et al., 2022). As shown in Table 2, the HTMT values were all < 0.869, demonstrating satisfactory discriminant validity.

**Table 2:** Discriminant validity of constructs based on the HTMT ratio.

Construct	Continuance intention	Perceived ease of use	Perceived enjoyment	Well-being
Continuance intention				
Perceived ease of use	0.653			
Perceived enjoyment	0.523	0.705		
Well-being	0.648	0.869	0.748	

### 4.2 Structural model assessment

**Figure 2:**
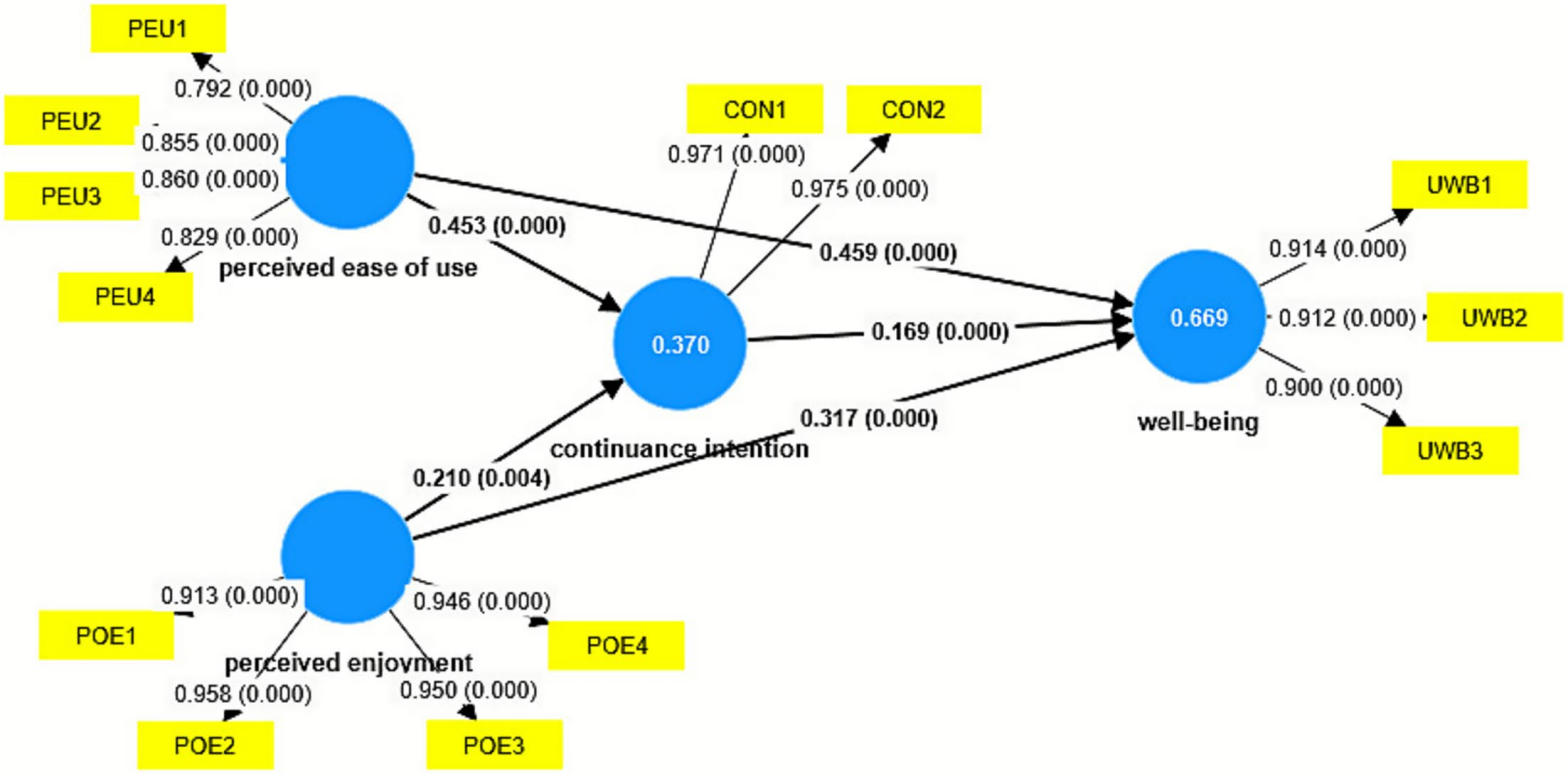
Bootstrapping results of the structural model in SmartPL.

The structural model was assessed by bootstrapping and blindfolding using SmartPLS software. The results are presented in Table 3 and Figure 1. In Figure 1, the outer model shows the outer loading and *p*-value, whereas the inner model shows the β-value and p-value. R^2^ is the dependent variable. The results of the path coefficient analysis using SmartPLS (Table 3; Figure 2) show significant relationships among the constructs. In Figure 2, the inner model illustrates the β-value and p-value, the outer model displays the outer loading and p-value, and the construct shows the R^2^ value. Perceived ease of use strongly influences both continuance intention (β = 0.453, t = 7.150, *p* < 0.001, f^2^ = 0.193) and well-being (β = 0.459, t = 8.263, p < 0.001, f^2^ = 0.316), indicating a robust positive effect size (Venkatesh et al., 2003). Perceived enjoyment positively affects continuance intention with a small effect (β = 0.210, t = 2.918, *p* = 0.004, f^2^ = 0.041) and well-being with an effect (β = 0.317, t = 6.071, p < 0.001, f^2^ = 0.173). The direct relationship between continuance intention and well-being is also statistically significant (β = 0.169, t = 3.670, p < 0.001), but the effect size is small (f^2^ = 0.054) (Li et al., 2025). Furthermore, mediated effects were established, with perceived ease of use leading to well-being through continuance intention (β = 0.076, t = 3.424, *p* = 0.001) and perceived enjoyment following a similar path (β = 0.035, t = 2.105, *p* = 0.035). Correspondingly, perceived ease of use had a strong total effect of 0.535 (direct effect β = 0.459 + indirect effect β = 0.076), while perceived enjoyment had a moderate total effect of 0.352 (direct effect β = 0.317 + indirect effect β = 0.035) (Hair et al., 2022).

**Table 3:** Structural model results: path coefficients, effect sizes, and predictive relevance.

Path	β	*t*	*p*	f^2^
Continuance intention → well-being (H 1)	0.169	3.67	0.000	0.054
Perceived ease of use → continuance intention (H 2)	0.453	7.15	0.000	0.193
Perceived ease of use → well-being (H 3)	0.459	8.263	0.000	0.316
Perceived enjoyment → continuance intention (H 4)	0.210	2.918	0.004	0.041
Perceived enjoyment → well-being (H 5)	0.317	6.071	0.000	0.173
Perceived ease of use → continuance intention → well-being	0.076	3.424	0.001	
Perceived enjoyment → continuance intention → well-being	0.035	2.105	0.035	
	R^2^	Q^2^		
Continuance intention	0.370	0.344		
Well-being	0.669	0.546		

The R^2^ value represents the coefficient of determination, which measures the amount of variance in the dependent variable that can be explained by the independent variables in the model. In this analysis, R^2^ values for continuance intention (0.370) indicate that 37% of its variance could be explained by perceived ease of use and perceived enjoyment, indicating moderate explanatory power. The R^2^ value for well-being (0.669) indicates that up to 66.9% of the variance in well-being was explained by perceived ease of use, perceived enjoyment, and continuance intention. This signifies substantial explanatory power within the model.

However, the Q^2^ value is a measure of the model’s predictive relevance. Q^2^ values above zero suggest that the model has predictive relevance for the dependent variables (Li et al., 2025). In this study, the Q^2^ values for continuance intention (0.344) and well-being (0.546) indicated good predictive relevance, demonstrating the effectiveness of the model in predicting the data.

## 5 Discussion

This study employed PLS-SEM to explore the predictive role of perceived ease of use, perceived enjoyment, and continuance intention as an extended TAM that influences medical students’ well-being in a hybrid learning environment. As all hypotheses were accepted, this study further corroborated the robustness, flexibility for extensions, and explanatory power of the TAM (Attié and Meyer-Waarden, 2020). Our results also advance the literature by validating well-being as a direct outcome within an extended TAM framework that has not yet been systematically tested in the context of medical education. While prior studies typically emphasize academic achievement or satisfaction, this research demonstrates that ease of use and enjoyment contribute to psychological well-being through continuance intention. This extension highlights the dual role of hybrid learning technologies, not only as instructional tools but also as protective resources for students’ mental health. By situating well-being at the center of the TAM, this study provides a novel theoretical integration of health-oriented perspectives, bridging educational technology and medical education research.

The significant relationship between perceived ease of use and students’ well-being (H1) suggests that user-friendly interfaces can effectively promote positive educational outcomes by enhancing students’ well-being. This finding aligns with research in the fields of smart-connected objects (Attié and Meyer-Waarden, 2020), e-learning (Alqahtani et al., 2022; Ficapal-Cusí et al., 2024), and online learning (Xiao et al., 2023). Among these variables, perceived ease of use emerged as the strongest predictor of medical students’ well-being, highlighting its critical role in educational technology, particularly because technological complexity is the main challenge students encounter in blended learning (Rasheed et al., 2020).

By confirming that perceived enjoyment predicts students’ well-being (H2), our findings echo those of e-learning research by Frederick and Lazzara (2020) and Ficapal (Ficapal-Cusí et al., 2024). The current study extends this insight into hybrid learning environments, suggesting that enjoyment derived from hybrid formats can substantially enhance students’ well-being. However, additional research is necessary to differentiate hybrid environments from strictly online or in-person formats to understand the causal links and processes via which enjoyment influences well-being.

Further, the study confirms that continuance intention positively influences well-being (H3), supporting the findings in online settings (Linares et al., 2021; Lu et al., 2019). However, the effect size of this path was small (f^2^ = 0.054), indicating that, although statistically significant, its practical impact may be limited compared to perceived ease of use. This implies that students who consistently use hybrid learning platforms may experience additional benefits for their well-being, but other factors—particularly perceived ease of use and enjoyment—play a more substantial role in shaping their overall psychological experience with hybrid learning. This means that continuance intention should be seen as one of several contributing factors rather than a dominant determinant.

Supporting the key TAM assumption, perceived ease of use significantly predicts continuance intention (H4). This result is pivotal for understanding how usability impacts long-term engagement with technology, as noted in diverse technological studies (Sun, 2024; Kim and Song, 2022). This finding contrasts with previous studies conducted in other contexts that reported no significant effect (Linares et al., 2021; Han and Sa, 2022). A possible explanation for the different research findings could be attributed to the differences in participants’ characteristics and research context. For instance, one study explored Korean university students’ learning experiences in online classes (Han and Sa, 2022), whereas another focused on players’ experiences in massive multiplayer online games (Linares et al., 2021). In contrast, this study focused on medical students’ experiences in hybrid learning environments. These differences highlight the necessity for technology developers to consider user-specific needs and contexts when creating instructional technologies.

Additionally, in line with studies emphasizing the importance of affective elements in academic persistence (Rodrigues et al., 2020), this study found that perceived enjoyment was a strong predictor of continuing intention (H5). This indicates that medical students are more inclined to stick to a hybrid learning environment if they find it enjoyable.

The significant but small indirect effects via continuance intention indicate a pattern of partial and relatively weak mediation: continuance intention transmits some of the influence of perceived ease of use and enjoyment on well-being, but most of the variance in well-being is still explained by direct paths from these beliefs. This suggests that mechanisms other than continued system use are likely to play a substantial role in linking students’ technology-related beliefs to their well-being. Alternative explanations and potentially confounding variables should be considered. For example, students with higher pre-existing well-being, better prior academic achievement, or more supportive learning environments (e.g., teacher support, peer relationships, or lighter workload) may be more likely to perceive hybrid learning systems as easy to use and enjoyable and to continue using them. Personality traits, such as resilience or grit, could also jointly influence both technology-related perceptions and well-being.

## 6 Conclusion

The results of this study contribute to a broader understanding of how hybrid learning environments can support the well-being of medical students and promote positive educational outcomes in hybrid learning. The results indicated that perceived ease of use and perceived enjoyment positively affected continuance intention, which, in turn, enhanced the well-being of medical students. These findings support the robustness, flexibility of extensions, and explanatory power of the TAM. However, the comparison between perceived ease of use and perceived enjoyment revealed that students placed greater weight on the ease of use of the interface, as evidenced by its strong effect size on well-being compared to the moderate effect of perceived enjoyment. This suggests that the functionality and user-friendliness of a hybrid learning platform are more critical to students than enjoyment. These findings support the body of knowledge on technology adoption and go beyond earlier studies by highlighting user-friendly interfaces and enjoyable learning experiences in hybrid environments (Xiao et al., 2023).

### 6.1 Implication

To improve the well-being of medical students in hybrid learning environments, medical educators must prioritize the ease of use of hybrid learning platforms and strive to create engaging and interactive content that stimulates interest and enjoyment (Azizi et al., 2020). This understanding guides instructional design, technological integration, and student support services. By prioritizing intuitive and simple designs and enjoyable user experiences, medical educational institutions can work closely with developers to ensure that hybrid learning tools meet their needs, thereby enhancing students’ learning experience and well-being. Additionally, providing adequate technical support and training for both medical students and educators is essential for addressing the challenges of hybrid learning (Jebraeily et al., 2020).

### 6.2 Limitations

This study had several limitations. The use of self-reported questionnaires may have introduced a response bias, thereby undermining the validity of the results. Additionally, the generalizability of the results is limited, as the study was conducted at only three medical universities in Guangxi, China, which may not reflect the experiences of medical students elsewhere. The cross-sectional design also restricts the ability to observe changes in the collected data over time and, therefore, limits causal inference. In particular, the cross-sectional nature of the data supports correlational associations but does not allow strong causal inferences to be drawn; thus, reverse or reciprocal relationships cannot be ruled out. Moreover, the observed relationships may have been influenced by unmeasured confounding variables. In addition, unmeasured variables, such as students’ pre-existing mental health, perceived academic workload, and support from family and peers, may also shape well-being in hybrid learning and were not controlled for in this study. For a more comprehensive understanding, future studies should incorporate qualitative methodologies, expand geography, employ longitudinal designs, and include additional control variables to account for potential confounders.

## Data Availability

The datasets presented in this article are not readily available because they contain information about human participants and are subject to institutional ethical and privacy restrictions. Requests to access the datasets should be directed to the corresponding author.

## Appendix

**Table:**

Variable	Coding	Item
Continuance Intention	CON1	I intend to continue using hybrid learning in the future.
	CON2	I plan to continue using hybrid learning.
Perceived Enjoyment	POE1	Using hybrid learning is enjoyable.
	POE2	I have fun while using the hybrid learning.
	POE3	I experience excitement when using hybrid learning.
	POE4	The hybrid learning is pleasant.
Perceived Ease of Use	PEU1	My interaction with the hybrid learning system is clear and understandable.
	PEU2	Interacting with the hybrid learning system does not require a lot of my mental effort.
	PEU3	I find the hybrid learning system to be easy to use.
	PEU4	I find it easy to get the hybrid learning system to do what I want it to do.
Well-Being	UWB1	I am satisfied with my current academic achievements in hybrid learning.
	UWB2	I am satisfied with my current study situation in hybrid learning.
	UWB3	I am satisfied with my general study conditions in hybrid learning.
